# Home Quick – Occupational Therapy Home Visits Using mHealth, to Facilitate Discharge from Acute Admission Back to the Community

**DOI:** 10.5195/ijt.2017.6218

**Published:** 2017-06-29

**Authors:** JACQUELINE NIX, TRACY COMANS

**Affiliations:** 1 OCCUPATIONAL THERAPY DEPARTMENT, REDCLIFFE HOSPITAL, METRO NORTH HOSPITAL AND HEALTH SERVICE DISTRICT, ANZAC AVE, REDCLIFFE, 4020, AUSTRALIA; 2 METRO NORTH HOSPITAL AND HEALTH SERVICE, BUTTERFIELD ST, HERSTON, 4006, AUSTRALIA; 3 MENZIES HEALTH INSTITUTE QUEENSLAND, GRIFFITH UNIVERSITY, NATHAN, 4111, AUSTRALIA

**Keywords:** Acute discharge, Home assessment, mHealth, Mobile technology, Occupational therapy

## Abstract

This article reports upon an initiative to improve the timeliness of occupational therapy home visits for discharge planning by implementing technology solutions while maintaining patient safety. A community hospital in Queensland, Australia, hosted a process evaluation that examined which aspects of home visiting could be replaced or augmented by alternative technologies. Strategies were trialled, implemented and assessed using the number of home visits completed and the time from referral to completion as outcomes. A technology-enhanced solution called “Home Quick” was developed using technology to facilitate pre-discharge home visits. The implementation of Home Quick resulted in an increase in the number of home visits conducted prior to discharge (50% increase from 145 to 223) and significantly increased the number of patients seen earlier following referral (*X*^2^=69.3; p<0.001). The substitution of direct home visits with technology-enabled remote visits is suitable for a variety of home visiting scenarios traditionally performed by occupational therapists.

Occupational therapists (OTs) are often required to assess a patient’s home for safety before discharge as part of their role in acute care and rehabilitation teams (American Geriatrics Society/British Geriatric Society, 2011; Cumming et al., 2001; Cumming et al., 1999). These typically take the form of an OT and an OT assistant attending the patient’s home, usually with family in attendance, to assess the person-environment fit, and ensure a safe discharge. These home visits have been shown to minimise the risk of falls and other adverse events (Cumming et al., 1999; Di Monaco et al., 2008; Peterson & Clemson, 2008; Pighills, Torgerson, Sheldon, Drummond, & Bland, 2011). A complex home assessment with a patient including driving and administrative tasks can take over five hours.

When the OT is unable to visit the home in a timely manner and the patient is considered medically stable and ready for discharge, a subsequent delay to discharge occurs that is commonly termed “bed blocking” (McBride, 1995). The problem is further magnified when the time spent conducting a home visit delays the treatment of other inpatients under the care of the OT. This can result in precipitous discharges that increase the risk of early readmission (Dobrzanska & Newell, 2006). It is well recognised that patients recuperating in acute facilities under current health system constraints is not a viable practice. Demand simply outweighs capacity (Travers et al., 2008). Therefore, it is important that prompt home visits can be performed in order to facilitate the safe and timely discharge of appropriate patients.

The World Federation of Occupational Therapists has published a position statement on the use of telehealth to improve accessibility to occupational therapy (World Federation of Occupational Therapists, 2014). Telehealth is considered appropriate when in-person services are not feasible or optimal for delivering care, and/or both provider and client are happy with provision by telehealth. Telehealth has been suggested as an effective and reliable way to access home modification services (Cason, 2014; Sanford, Jones, Daviou, Grogg, & Butterfield, 2004). The rapidly expanding opportunities and funding for telehealth provision are creating a space for occupational therapists to deliver services including home assessments and modification recommendations in new ways (Cason, 2012).

Published use of technology for home visits is limited and has mainly been focussed on rehabilitation. One study used photography to identify potential hazards remotely, and follow up with a conventional home visit if indicated (Daniel et al., 2013). A comparative trial of pre-admission visits for patients undergoing joint replacements found that there was high agreement between in-home and remote OT assessments with consistency in 90% of the identified home environment hazards and height measurements only varying between 0.1–3.3 cm (Hoffmann & Russell, 2008). This study used a conventional internet connection with videoconferencing facilities. However, these conventional telehealth systems are not necessarily suitable for the requirements of acute wards as they require pre-booking and specialist technical knowledge to operate. Mobile technology offers an alternative that is relatively cost-effective and easily accessible to both patients and OTs.

## BACKGROUND TO THE ‘HOME QUICK’ PROJECT

The Metro North Hospital and Health Service (MNHHS) covers a population of approximately 900,000 in the southern part of Queensland, Australia. The district contains five public hospitals, two tertiary hospitals located in the capital Brisbane, two community hospitals covering outer metropolitan areas and one small rural hospital. This project was located in the catchment of the community and rural hospitals and was based in Redcliffe. The area has a high elderly population and low socio-economic status with the highest concentration of older lone person households in Australia according to census data (Australian Bureau of Statistics, 2004).

There were a number of issues that arose to subsequently drive the need to investigate the use of technology to improve home visiting services:

- The OTs at the community and rural hospitals covered 80% of the geographic area of MNHHS (4157 km^2^).
- The large area resulted in frequent 1–2 hour (one way) trips by car to the patient’s home. The combination of requirements to conduct the home assessment, administrative tasks and report writing means a home visit could take over half the working day.
- The community and rural hospitals have a small workforce to patient ratio in the acute wards (community hospital one has 120+ beds with 3.0 Full Time Equivalent (FTE) OT staff; community hospital two has 70+ beds with 2.0 FTE; and rural hospital has 10 beds with 0.2 FTE)
- There was a loss of public federally funded community health OTs in 2010–11 due to the restructuring of services by the state government, with OT services not fully replaced.
- Risk assessments could stop a home visit from being conducted. For example: floods; bad weather; distance; dangerous animals on premises; not able to take another staff member; patient at risk of absconding.
- Car availability, family availability, distance to travel, time to organise equipment or rails if required, and competing demands on wards limited the ability of OTs to rapidly perform home assessments required for discharge.

At the same time, personal technologies such as smart phones were becoming ubiquitous and communication applications (apps) were readily available that had the potential for use in home visiting scenarios. In addition, routinely collected data systems in MNHHS were accessible for the purposes of evaluation. For example, being able to scan activity in real time with meaningful units linked to the Health Round Table Data, a database which is used to compare peer facilities to examine efficiency and identify gaps in services to be investigated (The Health Roundtable, 2015). The aim of this project was to improve the timeliness of home visits by implementing and using technology-based solutions while maintaining patient safety.

## MATERIALS AND METHODS

This study used the “Plan, Do, Study, Act” (PDSA) framework for the implementation and evaluation of this project. PDSA is a widely accepted method in health care for quality improvement activities allowing small scale experimentation before implementing more broadly (Taylor et al., 2014). In brief, the components used in this study were:

- Plan: conduct literature review; conduct survey of patients and staff; compare different phones and laptops; understand legal and ethical implications.
- Do: create new model and trial with orthopaedic patients.
- Study: evaluate the process and change resources where needed.
- Act: implement for a trial period.?

### LITERATURE SURVEY

A review of the literature was undertaken by searching PubMed, CINAHL and the Clinician’s Knowledge Network with the key words: home visit, occupational therapy, assessment and technology. A small number of articles were found discussing the use of telehealth in occupational therapy (summarised earlier) and no relevant articles were found that had used mobile technology for home visiting or were able to inform the proposed model of care.

### SCOPING SURVEY

An initial survey of older patients was carried out to determine whether the target population had access to smart phones and relevant video calling apps such as Skype. Included participants were patients from inpatient medical and surgical wards aged over 70 years who were able to provide informed consent. Patients were excluded if they had previously experienced a stroke or were currently experiencing other cognitive issues such as dementia or delirium. For people with non-English speaking backgrounds, an interpreter was used where required.

### MODELS OF CARE

A process evaluation of home visiting tasks was undertaken with the OT departmental staff at Redcliffe hospital to identify tasks that: (a) should not change; (b) could not change; and (c) should and could change with the addition of technology. Following this, a focus group session was conducted with 15 staff members (12 OTs, 2 OT assistants, 1 line manager). The purpose of this session was to problem solve a range of viable options (both technology-driven and usual care) to include in the implementation trial with patients awaiting discharge from hospital and develop a risk screening tool that could identify issues with home visiting that may preclude particular types of options for certain patients.

During home visits undertaken by OT staff, measurements are routinely taken within the home of key distances such as the width of doorframes, the height of steps, and the height of toilets. In order to facilitate the trial of technology-assisted home visits, a booklet was designed for patients, families, carers and new staff members to instruct them on the correct way to take these measurements. The success of the instruction booklet was evaluated by comparing measurements taken on the same properties by: (a) OT staff; and (b) a convenience sample of patients awaiting hip and knee replacement surgery who were members of a consumer advocacy group.

### IMPLEMENTATION TRIAL

The process was initially piloted on pre-operative orthopaedic patients awaiting joint replacement surgery, with the exclusion of patients from residential care facilities. Following the pilot, the process was expanded to include any patient requiring home assessment to ensure safe discharge, prevent complications and /or readmission. The range of options (“Home Quick”) was trialled from September to December 2013, and implemented for all suitable patients from February 2014.

## DATA ANALYSIS

A pre-post analysis was conducted of the intervention to understand the changes in throughput and efficiency gained from the introduction of the use of technology. Data on the days taken to complete home assessments from referral, increases in ward activity by OT and increases in home assessments was collated using routinely collected administrative data from electronic data systems.

Data was collected in two six month periods; pre-intervention was collected Feb to Aug 2013 and post intervention Feb to Aug 2014. The same months of the year were chosen so that seasonal variations in home visit requests and other acute ward demands would be similar and thus accounted for. The intervening 6 months allowed the new service delivery model to be piloted and subsequently embedded into practice.

As the process was implemented as a quality improvement activity of an existing service, human research ethics approval was not required.

## RESULTS

### SCOPING SURVEY

Thirty patients awaiting orthopaedic surgery were recruited from either the orthopaedic ward or medical ward and gave consent to participate in the survey. Twenty-seven (90%) patients owned a smart phone, and of these patients, 20 (75%) were aware of Skype or video phone calling and 13 (50%) had used Skype on a computer (with and without help). Of the people who did not have a smart phone (n = 3, 10%), all of them had a next of kin who had a smart phone. Only 4 (13%) of the 30 patients felt uncomfortable with the idea of a video call showing the inside of their home.

### MODELS OF CARE

The standard home visit procedure used a pen, paper, tape measure and standard digital camera. An area for improvement would be that photographs and drawings of the room would be made where the equipment (e.g., grab rail) should be placed. Following the visit, details of the home visit would need to be typed into a report with drawings and photographs imported. This would be printed and filed in the patient’s chart.

The results of the process evaluation are reported in Table 1. The OT department staff nominated a number of processes within standard care that could be changed or improved with technological solutions (e.g., the use of multiple paper forms used for recordkeeping during home assessments that required manual entry into a computer upon returning to the office). There were also aspects that could not be changed (e.g., the distance to a home assessment) that could potentially be mitigated with the use of a technology substitute to a traditional home visit.

During the focus group session, participants were enthusiastic about finding ways to eliminate duplication and be more administratively efficient using technology. The critical issue was removing the need for the OT to leave the hospital. Allied Health Assistants (AHAs) were already undertaking ‘access visits’ to photograph and measure appropriate areas of the house. The OT would then review this information on the AHA’s return to the hospital. To optimise this process, real time video footage would be ideal to allow the OT to view the different areas of the home in more detail, whilst still having a staff member on site to ask specific questions of the patient and their carers/family, for example, the slipperiness of the floors.

The first solution produced by the focus group session was an innovative mobile health (mHealth) process to enable the OT to talk to an AHA, junior OT (i.e., a new graduate, up to about five years post-graduation), or the carer/ family member at the house, and even pre-op patients using smart phones and readily available apps. The Hospital Management Committee approved the upgrade of staff mobile phones to a smart phone with a data plan in order to facilitate the implementation of this solution.

A second solution to save time in completing home visits involved moving from the standard home visit procedure with pen and paper record keeping to a laptop or tablet device for electronic record keeping. The device needed: (a) a USB port; (b) to be Microsoft Word® compatible; (c) to perform multiple functions such as still photography and video filming; and (d) to allow the OT to finalise the report at the patient’s home or during the drive back to the hospital. This process removed the duplication present in the standard home visit of re-entering data into a computer once back at the hospital.

The combination of options for technology enabled or enhanced visits constitutes the ‘Home Quick’ intervention (see Table 2). Some of the ‘Home Quick’ options were traditional visits augmented by technology whilst others were new mHealth options. These options recognise the need for some assessments that require an OT to attend the home of the patient, whilst promoting the option that many conventional visits could be replaced or improved with the use of technology.

**Figure 1:**
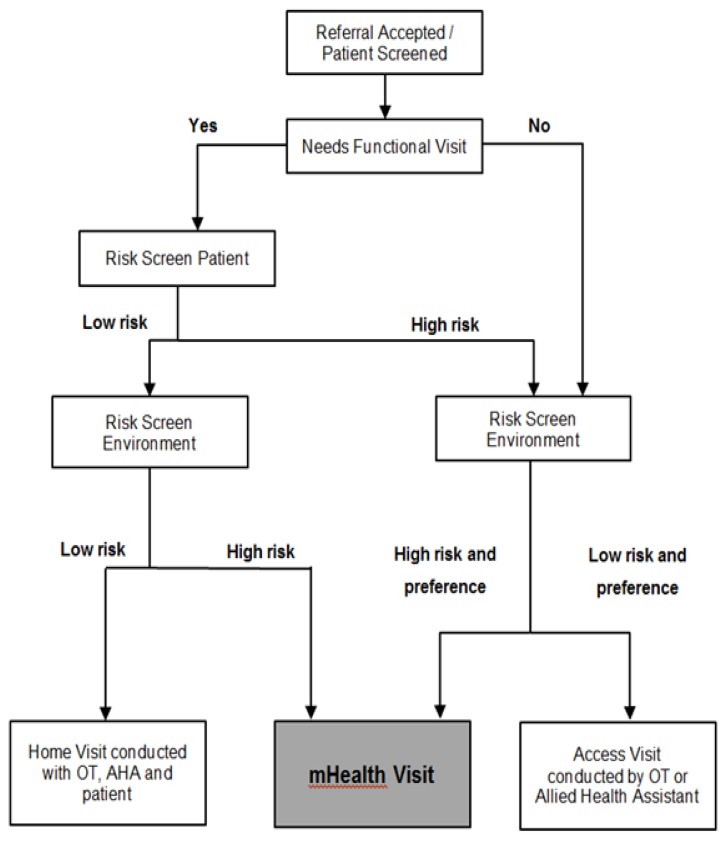
The risk screening tool developed during the pilot implementation phase.

The risk screening tool developed during the pilot implementation phase is described in Figure 1. Firstly, patients were assessed to determine if they would benefit from a functional home visit during which an OT would check the safety of the patient to navigate his/her home. If the patient required such a visit, the second step was to identify if the patient was medically safe to attend a home visit whilst still an inpatient. Thirdly, if the home environment was assessed as safe, an OT would then attend the home visit with the patient. If the patient was not medically safe enough to attend the home visit, an alternative access visit, either the traditional option with an OT or AHA visiting the home, or the technological mHealth option could be used.

The instruction booklet formed to educate patients, families, carers and new staff on the correct method of measuring a property was found to be useful and informative enough for family or assistants to take measurements. Only one out of the ten homes assessed was measured incorrectly in one area. As a result, revisions were made to target door jamb measurements and no further errors were found in a further six assessments.

### IMPLEMENTATION TRIAL

Table 3 presents the number of occupational therapy interventions conducted during both trial time periods. After the introduction of Home Quick, OTs were able to undertake 78 more home visits (145 vs 223), an increase of more than fifty percent. In addition, the number of other inpatient interventions (assessments and treatments) also increased (range, +16 to +115%). Overall, total interventions increased by 31% over the equivalent six months from the year before with the same level of staffing (3.0 FTE) in place. Staff were able to give patients on average an additional one occasion of service post introduction of the new service model.

Table 4 presents the percentage of home visits completed within specific time frames from the time of referral. Significantly more patients had referrals completed earlier following referral after the implementation of mHealth (*X*^2^=69.3; p<0.001). In particular, the number of patients seen on the same day or within one day of referral increased from 20% to 60%.

## DISCUSSION

This project has demonstrated that simple on-site home visits can safely be performed or augmented using technology, and that this service delivery model improves the throughput and efficiency of hospital-based OT departments without changing staffing levels. Home Quick reduced the time and administrative burden required to perform routine home visits. As a result, OT departments were able to improve their service capacity in other sectors of care. The implementation of the Home Quick intervention resulted in shorter wait times between referral and completion of home visits and an increase in the overall capacity of the OT workforce to undertake both home and other important assessments required in the acute sector. Home Quick has now been embedded into practice and is part of the standard home visiting options available within MNHHS.

Technology has the ability to substantially change how health care is provided. For the MNHHS in particular, there are specific advantages to being able to conduct home assessments remotely. Patients within the MNHHS are admitted from all over the state to tertiary hospitals in Brisbane to access specialised services. In these cases, the patient’s home may be over 1,000 km away from their tertiary facility and physical home visits are not possible. Other benefits include being able to complete a home visit in situations that would otherwise not permit a home visit. Such situations include where an environmental risk has been identified, such as a dangerous dog or difficult family situation. Technology is changing rapidly; smart phones and apps that allow video streaming are becoming more easily accessible, allowing this type of home visiting to become routine practice for OTs within MNHHS.

There were many challenges that had to be overcome in order to implement Home Quick successfully. Many of these involved legislative requirements and existing IT systems. Legislative issues arose around the inability to send data from home visits via email or to store it on cloud servers as these servers were outside the organisation and therefore did not comply with privacy regulations. Video calls could not be recorded live. This was overcome by the use of tablets that could store the data until it could be downloaded at the hospital. Policy around use of technology within health care organisations can be a barrier to its implementation, delaying the use of such devices and limiting efficiency. Internet access proved to be another challenge: there was limited Wi-Fi and internet coverage within the hospital, and some patients had limited data on their mobile plans, increasing the cost of their participation. Finally, not all OTs were comfortable with the use of technology to facilitate home visits, perceiving the change as a challenge or a diminishing of the role of the OT.

The quantitative improvements in this project may not all be attributable to Home Quick. In the health service district at the time, there was a focus from the health service on improving patient flow and discharge patients home safely and quickly. A concurrent project was the use of a discharge planning co-ordinator to facilitate earlier discharge. Consequently, we are unable to separate definitively the improvements resulting from Home Quick from these other efficiency initiatives. However, the mHealth options developed in this project are a considerably faster way of conducting a home visit compared to the traditional physical home visit. Furthermore, our practising clinicians perceive value in this service, demonstrated by the way staff have now embedded Home Quick into standard practice at the hospitals within our service district.

## CONCLUSION

Through the use of the “Plan, Do, Act, Study” framework, a technology-assisted service delivery model (Home Quick) for home visits by occupational therapists was successfully implemented in three community and rural hospitals. This led to an overall increase in the productivity of OT staff, as well as improved timeliness of OT assessment for suitable patients. This can serve as a model for other OT departments to consider the use of technology to improve productivity in their departments, with appropriate consultation with their health care organisation to ensure flexibility and sustainable uptake of a technology used widely by the general community.

## Figures and Tables

**Table 1:** Process Review Outcomes

Level of change	Task
Should not change	Clinical time with patient in home for complex patients, complex homes or complex social situations
Could not change	Distance to house and back again High risk home environments Out of catchment
Should & could change	Administrative time taken using paper and pen to draft report, draw diagrams and then transfer onto computer based diagrams and word processed reports. Carrying multiple objects – phone, camera, video camera, tape measure, airport bag with paperwork, book of maps Use of drive time Repeated home visits

**Table 2:** Home Quick Options

Type	Description	Patient present	Family present	Staff	Technology role in home visit
**Functional**	Onsite Home Visit	Yes	Usually	OT and AHA	Augmented using tablet and smart phone
**Access**	Onsite Access Visit	No	Usually	OT or AHA	Augmented using tablet and smart phone
**m-Health**	Virtual Home Visit	No	Yes	OT present remotely	Using Smart Phone
**Family**	Home Assessment	No	Yes	With or without OT present remotely	OT at hospital as support on m-health

*Note*. AHA = Allied Health Assistant; OT=Occupational Therapist.

**Table 3:** Number of Occupational Therapy Interventions on Acute Inpatient Wards

Type of Intervention	Feb – Aug 2013	Feb – Aug 2014	Difference
**Home visits**			
Homes assessed by OTs*	145	223	+53%
**Other inpatient interventions**			
Initial Assessment	1285	1497	+16%
Self Care & Physical	1669	2611	+56%
Cognitive & Psychosocial	190	409	+115%
Total Interventions	3289	4740	+31%
Number of patients seen by OT	1177	1202	+0.02%
Occasions of service per patient	2.8	3.9	+39%

* does not include any OT assistant only access visits

**Table 4:** Time Taken to Complete the Home Visit from Time of Referral

Time to complete	Feb – Aug 2013	Feb – Aug 2014*
Same day	1%	24%
Within one day	19%	36%
Two days	37%	24%
Three or more days	43%	16%

* significant difference between time periods (*X*^2^=69.3; p<0.001).
